# Disparities in Peripheral Artery Disease-related Mortality in Chronic Inflammatory Disease in the United States from 1999 to 2020

**DOI:** 10.2174/011573403X353038241125050631

**Published:** 2024-12-10

**Authors:** April Olson, Hoang Nhat Pham, Ramzi Ibrahim, Mohammed Salih, Amitoj Singh, Mamas A. Mamas

**Affiliations:** 1 Department of Medicine, University of Arizona Tucson, Tucson, AZ 85721, USA;; 2 The Heart Hospital - Baylor University Medical Center, Plano, TX 75093, USA;; 3 Keele Cardiovascular Research Group, Keele University, Keele, Newcastle-under-Lyme, UK

**Keywords:** Peripheral artery disease, chronic inflammatory disease, chronic inflammatory, black populations, demographic data, atherosclerotic cardiovascular disease

## Abstract

**Introduction:**

Peripheral arterial disease (PAD) is a marker of significant atherosclerotic cardiovascular disease and is associated with greater healthcare burden and worse prognosis in individuals with chronic inflammatory disease (CID). We aimed to investigate temporal trends and disparities of PAD-related mortality in populations with CID from 1999-2020 across six common CIDs (*i.e*., chronic viral hepatitis, human immunodeficiency virus, inflammatory bowel disease, psoriasis, rheumatoid arthritis, and systemic lupus erythematosus).

**Methods:**

United States (US) PAD and CID-related mortality and demographic data from 1999-2020 were extracted from the CDC database through the multiple-cause-of-death files. Age-adjusted mortality rates (AAMR) per 1,000,000 and 95% confidence intervals were standardized to the 2000 US population. The mortality trends were analyzed using Joinpoint Regression.

**Results:**

A total of 22,175 PAD-related deaths were recorded in the population with CID between 1999 and 2020. Mortality remained stable during the 22-year period (AAPC -0.04%, *p=*0.95) with a cumulative AAMR of 4.64. Mortality was highest in rural counties (AAMR 5.27), and among non-Hispanic Black populations (AAMR 7.06). Among the CID subtypes, PAD mortality was highest in populations with RA (AAMR 2.48) and lowest in populations with psoriasis (AAMR 0.11).

**Conclusion:**

Our findings highlight the disparities of PAD mortality in patients with CID, with the Black population and rural communities disproportionately affected. Further investigation with individual-level data is warranted to identify the contributing factors for the observed disparities.

## 1 INTRODUCTION

Peripheral artery disease (PAD) remains a public health challenge, with prevalence of 5% in the United States (US) [1]. Chronic inflammatory disease (CID) is a risk factor for atherosclerosis and the development of PAD. Despite the decrease in PAD mortality in the general population in the past 20 years, longitudinal PAD mortality trends in the setting of CID are not well-documented [2]. Understanding these trends provides targets for resource allocation and tailored interventions. We aimed to investigate disparities in PAD-related mortality among different demographic groups and six common CIDs, *i.e.,* chronic viral hepatitis, human immunodeficiency virus (HIV), inflammatory bowel disease (IBD), psoriasis, rheumatoid arthritis (RA), and systemic lupus erythematosus (SLE) in the US [3].

## 2 MATERIALS AND METHODS

US mortality data from 1999 to 2020 were obtained from the multiple-cause-of-death files [4]. This analysis specifically utilized International Classification of Diseases, Tenth Revision (ICD-10) codes, in any position on a death certificate, PAD, and CID, including chronic viral hepatitis, HIV, IBD, psoriasis, RA, and SLE, in patients ≥25 years of age (Table **1**) [3]. Age-adjusted mortality rates (AAMR) per 1,000,000 and their 95% confidence intervals were calculated using the Direct Methods by standardizing age-specific rates in a population of interest to the 2000 US population. This approach accounts for variations in the age structure, allowing for unbiased comparisons of mortality rates between subpopulations and over time. Cumulative AAMR between 1999-2020 and their 95% CIs, subsequently, were calculated by taking the weighted of the annual AAMR, with weights proportional to population size for each year [5]. The Joinpoint Regression program was utilized to estimate temporal trends in mortality through log-linear regression models [6]. The average annual percentage change (AAPC) was estimated by Monte-Carlo permutation tests, which were calculated as the average of the annual percentage changes (APC). Two-tailed t-tests were used to evaluate for significant changes in the APC with *p*-value < 0.05 indicating statistical significance. For comparative purposes, we also obtained overall PAD mortality in populations without known CID to evaluate for temporal shifts. Institutional Review Board approval was not obtained due to the deidentified nature of the publicly available data regarding non-living humans (Tables **S1** and **S2**).

## 3 RESULTS

**Fig. (1):**
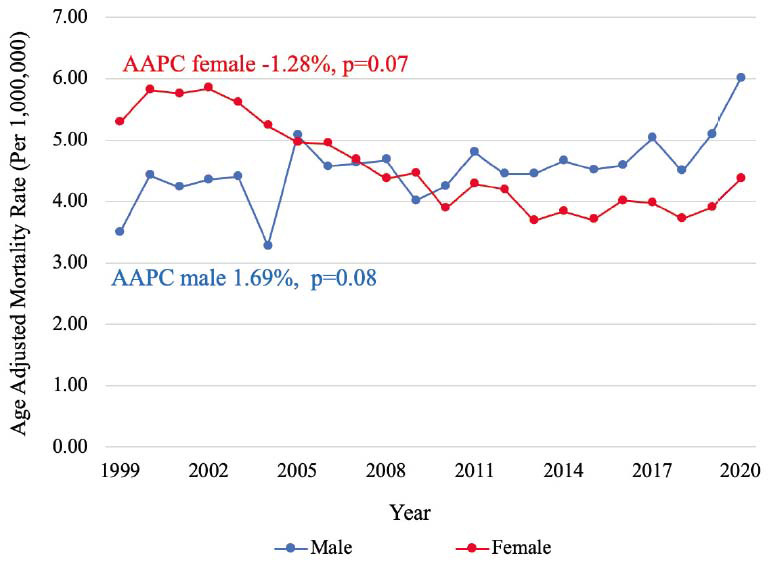
Mortality trends by sex. Trends in PAD mortality, stratified by sex, in populations with CID from 1999 to 2020. **Abbreviations:** AAPC=Average annual percentage change, CID=Chronic inflammatory disease.

**Fig. (2):**
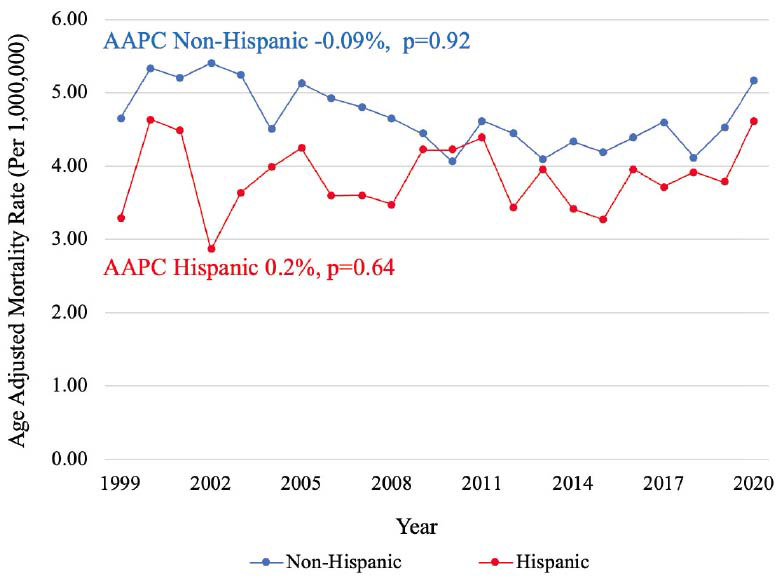
Mortality trends by ethnicity. Trends in PAD mortality, stratified by ethnicity, in populations with CID from 1999 to 2020. **Abbreviations:** AAPC=Average annual percentage change, CID=Chronic inflammatory disease.

**Fig. (3):**
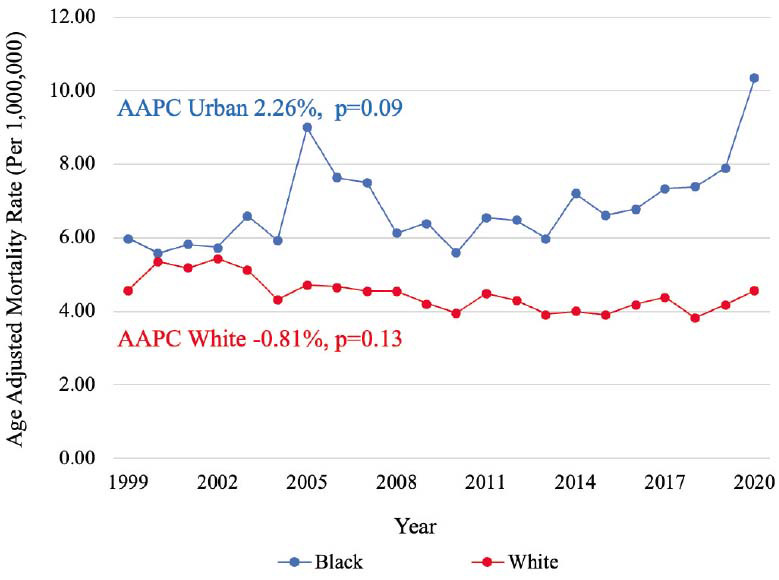
Mortality trends by race. Trends in PAD mortality, stratified by race, in populations with CID from 1999 to 2020. **Abbreviations:** AAPC=Average annual percentage change, CID=Chronic inflammatory disease.

**Fig. (4):**
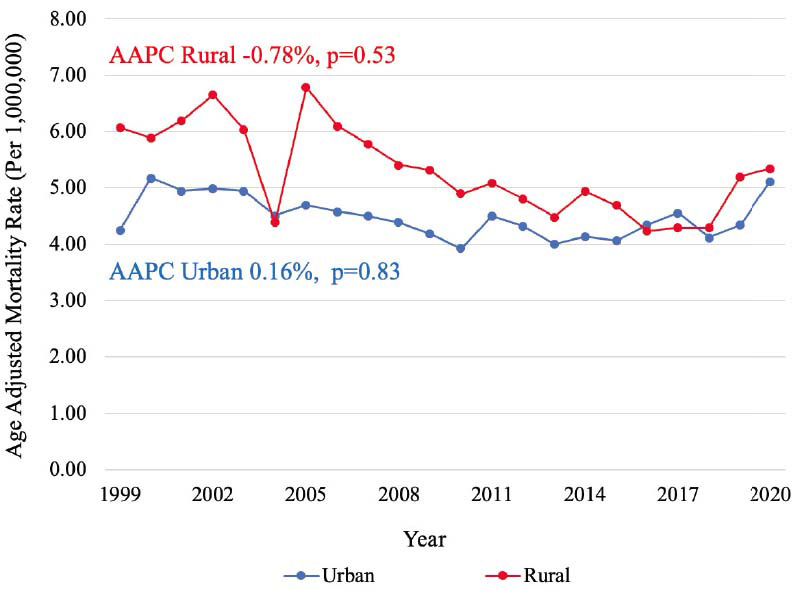
Mortality trends by urbanization. Trends in PAD mortality, stratified by urbanization, in populations with CID from 1999 to 2020. **Abbreviations:** AAPC=Average annual percentage change, CID=Chronic inflammatory disease.

Between 1999-2020, a total of 22,175 PAD-related deaths in the setting of CID were identified. PAD-related mortality in a population with CID remained stable during the past 22 years (AAPC -0.04%, *p=*0.95) with a cumulative AAMR of 4.64 [95% CI, 4.58-4.71], while PAD-related mortality in population without CID decreased consistently from 1999 to 2020 with an AAPC -2.71%, *p<*0.001 (Graphical abstract). In a population with CID, similar mortality rates were observed among males (AAMR 4.61 [95% CI, 4.52-4.70]) and females (AAMR 4.51 [95% CI, 4.43-4.59]) (Fig. **1**). Mortality was higher among non-Hispanic (NH) populations (AAMR 4.70 [95% CI, 4.64-4.77]) compared to Hispanic populations (AAMR 3.85 [95% CI, 3.65-4.06]) (Fig. **2**). Mortality was highest among NH Black (AAMR 7.06 [95% CI, 6.82-7.31] and NH American Indian/Alaska Native (AAMR 6.81 [95% CI, 5.79-7.83]) populations, followed by NH White (4.43 [95% CI, 4.36-4.50]) and NH Asian/Pacific Islander (AAMR 1.86 [95% CI, 1.66-2.07]) populations (Fig. **3**). Mortality was higher among rural regions (AAMR 5.27 [95% CI, 5.11-5.42]) compared to urban regions (AAMR 4.50 [95% CI, 4.44-4.57]) (Fig. **4**). Mortality trend analyses by other demographic factors and individual PAD causes are presented in Table **2** and Fig. (**5**).

Additionally, by specific CID stratification, RA (AAMR 2.48 [95% CI, 2.44-2.53]) had the highest mortality, followed by chronic viral hepatitis (AAMR 0.89 [95% CI, 0.87-0.92]), IBD (AAMR 0.47 [95% CI, 0.45-0.49]), SLE (AAMR 0.33 [95% CI, 0.31-0.35]), HIV (AAMR 0.33 [95% CI, 0.32-0.35]), and psoriasis (AAMR 0.11 [95% CI, 0.10-0.12]). Overall PAD AAMR related to RA was lower in 2020 (2.22 [95% CI, 2.04-2.40]) compared to 1999 (3.17 [95% CI, 2.91-3.43), largely due to the decreasing AAMR rates from 2001 to 2009 (APC -6.45, *p<*0.001) and 2009 to 2018 (APC -3.0, *p=*0.003). In contrast, the PAD AAMR in populations with CVH was higher in 2020 (1.51 [95% CI, 1.37-1.66]) compared to 1999 (0.13, 95% CI, 0.08-0.19]), mainly due to the uprising mortality from 2005 to 2020 (APC 3.67, *p<*0.001) (Table **3**) (Central Illustration).

## 4 DISCUSSION

Our findings revealed PAD mortality disparities in populations with CID consistently across demographic subgroups. PAD mortality disproportionately affected NH Black populations and rural communities. Among the CID subgroups, RA was associated with the highest PAD mortality, whereas psoriasis was associated with the lowest PAD mortality.

Despite the decrease in PAD mortality in the general population, the stability of PAD mortality in populations with CID is likely secondary to the confluence of divergent trends in PAD mortality across CID subtypes [2]. The decrease in PAD mortality among populations with RA could be attributed to the introduction of disease-modifying antirheumatic drugs (DMARDs) in the 1990s. There has also been a shift towards earlier initiation of DMARDs within the first few months of disease onset, aiming to prevent disease progression and inflammation, which also contributes to the sustained decline [7-9]. Despite a strong association with CVD, PAD-related mortality in SLE was surprisingly low in our analysis, especially compared to RA. This may be due to RA's continuous systemic inflammation accelerating atherosclerosis and increasing the incidence of fatal large vessel disease, while SLE is more often associated with fluctuating inflammatory burden and microvascular complications affecting the kidneys and skin [10-12].

Conversely, despite advancements in viral hepatitis therapy, such as the introduction of direct-acting antivirals, the increase in PAD mortality in individuals with CVH could be due to shifts from non-cardiovascular to cardiovascular deaths as treatments have changed the course of the disease process [3, 13]. As these therapies improve survival, patients with CVH live longer, reducing liver-related deaths (such as liver failure or infections) while increasing the likelihood of cardiovascular complications like PAD [14]. Individuals with CVH often have traditional cardiovascular risk factors such as smoking, diabetes, and dyslipidemia, which significantly contribute to PAD [15]. These risk factors, along with the under-diagnosis and under-treatment of PAD in this population, increase their PAD mortality. Additionally, the focus on managing liver-related issues may overshadow the need for cardiovascular screening and intervention since there are no policies on screening patients with chronic liver disease for PAD or cardiovascular disease. Moreover, by the time PAD is diagnosed, it is often in its late stages, making treatments like revascularization less effective and resulting in higher mortality [16]. The shift from liver-related to cardiovascular deaths may quickly become more evident, positioning PAD and other cardiovascular diseases as prominent causes of death for CVH patients as they live longer.

The higher PAD-related mortality observed in rural communities and non-Hispanic Black populations is likely multifactorial, including limited access to care, lack of healthcare resources, poor health literacy, and structural racism [17, 18]. Data from a 2011-2017 Surgical Quality database revealed racial disparities in PAD care, including higher incidence of amputations compared to limb-salvaging procedures [19].

This study has limitations, including the inability to establish causality, misclassification errors using ICD-10 codes, underestimation of PAD burden since PAD is often subclinical, and lack of patient-level data to adjust for potential residual confounding.

## CONCLUSION

Our analysis revealed that while PAD mortality has declined in the general population, this trend has not been mirrored in populations with CID. Black and rural populations have been disproportionately affected by higher mortality. These findings emphasize the need for future research utilizing individual-level data to better understand the factors driving these disparities. In response to these inequities, healthcare systems must allocate more resources to these vulnerable groups. This could include improving access to diagnostic tools such as the ankle-brachial index, as well as offering advanced treatments for PAD, including revascularization and amputation prevention strategies. Additionally, a more aggressive approach to managing modifiable risk factors-such as hypertension, smoking, and dyslipidemia—could significantly improve outcomes in these populations.

## Figures and Tables

**Fig. (5):**
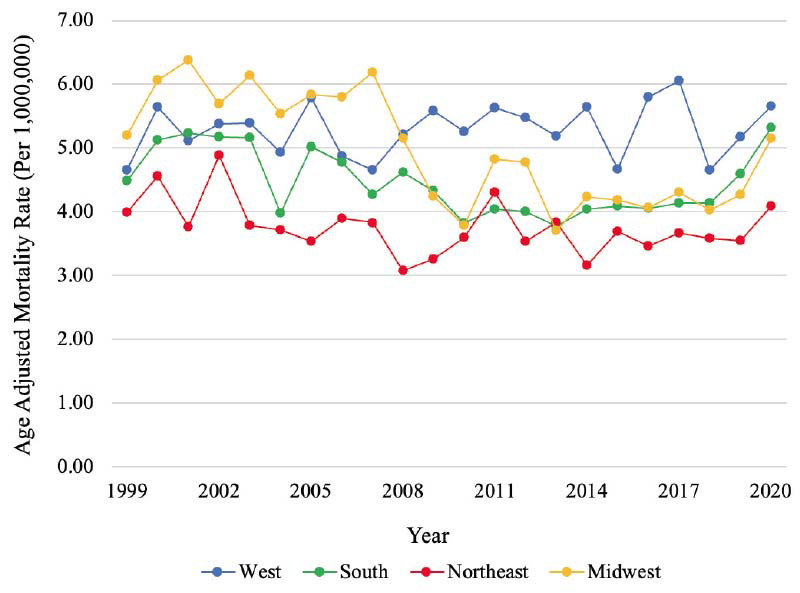
Mortality trends by US geographic census region. Trends in PAD mortality, stratified by US census region, in populations with CID from 1999 to 2020. **Abbreviations:** AAPC=Average annual percentage change, CID=Chronic inflammatory disease.

**Table 1:** International classification of diseases, tenth revision (ICD-10) codes.

**Disease**	**ICD-10 Codes**
Peripheral artery disease	E10.5, E11.5, E12.5, E13.5, E14.5, I70.x, I71.1–I71.6, I71.8–I71.9, I72.1–I72.4, I72.8–I72.9, I73.8–I73.9, I74.x, and I77.8–I77.9
Chronic viral hepatitis	B18
HIV	B20-B24
Inflammatory bowel disease	K50-K51
Psoriasis	L40
Rheumatoid Arthritis	M5–M6
Systemic Lupus Erythematosus	M32

**Table 2:** Joinpoint analysis by demographic characteristics. PAD mortality trends in population with CID in the US between 1999 and 2020.

**Joinpoint Segment**	**Years**	**APC [95% CI]**	**APC *p*-value**	**AAPC [95% CI]**	**AAPC *p*-value**
**Male**					
1	1999-2018	0.6344 [-0.15 - 1.42]	0.10	1.6853 [-0.21 - 3.62]	0.08
2	2018-2020	12.23 [-8.09 - 37.04]	0.24		
**Female**					
1	1999-2001	4.8621 [-8.37 - 20.01]	0.46	-1.28 [-2.65 - 0.11]	0.07
2	2001-2013	-3.70 [-4.60 - -2.78]	<0.001		
3	2013-2020	1.23 [-0.66 - 3.16]	0.18		
**Hispanic**					
1	1999-2020	0.20 [-0.67 - 1.07]	0.64	0.20 [-0.67 - 1.07]	0.64
**Non-Hispanic**					
1	1999-2018	-1.19 [-1.72 - -0.66]	<0.001	-0.09 [-1.60 - 1.45]	0.91
2	2018-2020	10.98 [-5.85 - 30.81]	0.20		
**Non-Hispanic White**					
1	1999-2015	-1.73 [-2.46 - -0.99]	<0.001	-0.81 [-1.85 - 0.24]	0.13
2	2015-2020	2.19 [-1.93 - 6.49]	0.28		
**Non-Hispanic Black**					
1	1999-2018	0.54 [-0.60 - 1.69]	0.34	2.26 [-0.37 - 4.96]	0.09
2	2018-2020	20.24 [-8.57 - 58.13]	0.17		
**Urban**					
1	1999-2018	-0.91 [-1.44 -0.37]	0.002	0.16 [-1.29 - 1.64]	0.83
2	2018-2020	10.91 [-5.20 - 29.77]	0.18		
**Rural**					
1	1999-2018	-2.10 [-2.92 -1.27]	<0.001	-0.78 [-3.19 - 1.69]	0.53
2	2018-2020	12.63 [-13.68 - 46.95]	0.36		
**Northeastern US Region**					
1	1999-2020	-0.62 [-1.34 - 0.09]	0.08	-0.62 [-1.34 - 0.09]	0.08
**Midwestern US Region**					
1	1999-2018	-2.51 [-3.44 -1.56]	<0.001	-1.06 [-3.83 - 1.80]	0.46
2	2018-2020	13.83 [-16.33 - 54.88]	0.39		
**Southern US Region**					
1	1999-2016	-1.72 [-2.47 - 0.96]	<0.001	-0.03 [-1.22 - 1.17]	0.96
2	2016-2020	7.47 [1.29 - 14.02]	0.02		
**Western US Region**					
1	1999-2020	0.25 [-0.33 - 0.82]	0.38	0.25 [-0.33 - 0.82]	0.38

**Abbreviations:** APC=annual percentage change, AAPC=average annual percentage change, CI=confidence interval, CID=chronic inflammatory disease.

**Table 3:** Joinpoint analysis. PAD mortality trends in population with CID in the US between 1999 and 2020.

**Joinpoint Segment**	**Years**	**APC [95% CI]**	**APC *p*-value**	**AAPC [95% CI]**	**AAPC *p*-value**
**All CID**					
1	1999-2018	-1.17 [-1.68 - -0.66]	<0.001	-0.04 [-1.44 - 1.37]	0.95
2	2018-2020	11.38 [-4.27 - 29.55]	0.15		
**Non-CID**					
1	1999-2001	7.00 [3.76 - 10.35]	<0.001	-2.71 [-3.15 - -2.27]	<0.001
2	2001-2010	-6.19 [-6.52 - -5.85]	<0.001		
3	2010-2018	-3.10 [-3.57 - -2.63]	<0.001		
4	2018-2020	5.87 [2.21 - 9.67]	0.004		
**Rheumatoid Arthritis**					
1	1999-2001	9.06 [-5.17 - 25.42]	0.20	-1.97 [-3.93 – 0.03]	0.053
2	2001-2009	-6.45 [-8.26 - -4.60]	<0.001		
3	2009-2018	-3.00 [-4.71 - -1.25]	0.003		
4	2018-2020	11.40 [-4.75 - 30.29]	0.16		
**Chronic Viral Hepatitis**					
1	2000-2002	-28.06 [-66.64 - 55.12]	0.37	11.95 [-3.17 - 29.39]	-0.13
2	2002-2005	120.76 [-12.98 - 460.06]	0.09		
3	2005-2020	3.67 [2.62 - 4.73]	<0.001		
**Inflammatory Bowel Disease**					
1	1999-2014	-3.30 [-4.75 - -1.83]	<0.001	-1.51 [-3.23 - 0.23]	0.09
2	2014-2020	3.10 [-2.37 - 8.88]	0.25		
**Systemic Lupus Erythematosus**					
1	1999-2010	-3.80 [-5.87 - -1.70]	0.001	-1.35 [-2.89 - 0.22]	0.09
2	2010-2020	1.42 [-1.24 - 4.16]	0.28		
**HIV**					
1	1999-2006	8.18 [1.76 - 15.01]	0.02	3.95 [-1.49 - 9.69]	0.16
2	2006-2009	-11.15 [-39.06 - 29.55]	0.51		
3	2009-2020	5.78 [3.41 - 8.20]	<0.001		

**Abbreviations:** APC=annual percentage change, AAPC=average annual percentage change, CI=confidence interval, CID=chronic inflammatory disease, PAD=peripheral artery disease.

## Data Availability

The authors confirm that the data supporting the findings of this research are available within the article.

## LIST OF ABBREVIATIONS

CID: Chronic Inflammatory Disease
DMARDs: Disease-modifying Antirheumatic Drugs
HIV: Human Immunodeficiency Virus
PAD: Peripheral Artery Disease
RA: Rheumatoid Arthritis
