# Supplementary material for: Disparities in Peripheral Artery Disease-related Mortality in Chronic Inflammatory Disease in the United States from 1999 to 2020
Source: Curr Cardiol Rev. 2024 Dec 10;21(3):E1573403X353038. doi: 10.2174/011573403X353038241125050631 (PMC12172234; doi:10.2174/011573403X353038241125050631)
Supplement: Supplementary file 1 [file CCR-21-3-E1573403X353038_SD1.pdf]

Supplementary Material

Disparities in Peripheral Artery Disease-related Mortality in Chronic Inflammatory Disease in the United States from 1999 to 2020

April Olson[1], Hoang Nhat[1,*], Ramzi Ibrahim[1], Mohammed Salih[2], Amitoj Singh[1] and Mamas A. Mamas[3]

[1]Department of Medicine, University of Arizona Tucson, Tucson, Arizona; [2]The Heart Hospital - Baylor University Medical Center, Plano, Texas; [3]Keele Cardiovascular Research Group, Keele University, UK

Supplement Table S1. International Classification of Diseases, Tenth Revision (ICD-10) codes

Disease	ICD-10 Codes
Peripheral artery disease	E10.5, E11.5, E12.5, E13.5, E14.5, I70.x, I71.1–I71.6, I71.8–I71.9, I72.1–I72.4, I72.8–I72.9, I73.8–I73.9, I74.x, and I77.8–I77.9
Chronic viral hepatitis	B18
HIV	B20-B24
Inflammatory bowel disease	K50-K51
Psoriasis	L40
Rheumatoid Arthritis	M5–M6
Systemic Lupus Erythematosus	M32

Supplement Table S2. Joinpoint Analysis by demographic characteristics. PAD mortality trends in population with CID in the US between 1999 and 2020.

Joinpoint segment	Years	APC [95% CI]	APC *p*-value	AAPC [95% CI]	AAPC *p*-value
Male					
1	1999-2018	0.6344 [-0.15 - 1.42]	0.10	1.6853 [-0.21 - 3.62]	0.08
2	2018-2020	12.23 [-8.09 - 37.04]	0.24		
Female					
1	1999-2001	4.8621 [-8.37 - 20.01]	0.46	-1.28 [-2.65 - 0.11]	0.07
2	2001-2013	-3.70 [-4.60 - -2.78]	<0.001		
3	2013-2020	1.23 [-0.66 - 3.16]	0.18		
Hispanic					
1	1999-2020	0.20 [-0.67 - 1.07]	0.64	0.20 [-0.67 - 1.07]	0.64
Non-Hispanic					
1	1999-2018	-1.19 [-1.72 - -0.66]	<0.001	-0.09 [-1.60 - 1.45]	0.91
2	2018-2020	10.98 [-5.85 - 30.81]	0.20		
Non-Hispanic White					
1	1999-2015	-1.73 [-2.46 - -0.99]	<0.001	-0.81 [-1.85 - 0.24]	0.13
2	2015-2020	2.19 [-1.93 - 6.49]	0.28		

1875-6557 /25 ©2025 Bentham Science Publishers

Non-Hispanic Black					
1	1999-2018	0.54 [-0.60 - 1.69]	0.34	2.26 [-0.37 - 4.96]	0.09
2	2018-2020	20.24 [-8.57 - 58.13]	0.17		
Urban					
1	1999-2018	-0.91 [-1.44 -0.37]	0.002	0.16 [-1.29 - 1.64]	0.83
2	2018-2020	10.91 [-5.20 - 29.77]	0.18		
Rural					
1	1999-2018	-2.10 [-2.92 -1.27]	<0.001	-0.78 [-3.19 - 1.69]	0.53
2	2018-2020	12.63 [-13.68 - 46.95]	0.36		
Northeastern US region					
1	1999-2020	-0.62 [-1.34 - 0.09]	0.08	-0.62 [-1.34 - 0.09]	0.08
Midwestern US region					
1	1999-2018	-2.51 [-3.44 -1.56]	<0.001	-1.06 [-3.83 - 1.80]	0.46
2	2018-2020	13.83 [-16.33 - 54.88]	0.39		
Southern US region					
1	1999-2016	-1.72 [-2.47 - 0.96]	<0.001	-0.03 [-1.22 - 1.17]	0.96
2	2016-2020	7.47 [1.29 - 14.02]	0.02		
Western US region					
1	1999-2020	0.25 [-0.33 - 0.82]	0.38	0.25 [-0.33 - 0.82]	0.38
Diabetic & PAD related mortality in CID					
1	1999-2020	0.25 [-0.83 - 1.34]	0.64	0.25 [-0.83 - 1.34]	0.64
Aortic aneurysm & PAD related mortality in CID					
1	1999-2020	-0.02 [-1.06 - 1.04]	0.97	-0.02 [-1.06 - 1.04]	0.97

Abbreviations: APC=annual percentage change, AAPC=average annual percentage change, CI=confidence interval, CID=chronic inflammatory disease.
